# Frailty increases vulnerability to postoperative cognitive dysfunction following general anesthesia in aged rats

**DOI:** 10.7150/ijms.139225

**Published:** 2026-09-03

**Authors:** Eun-Hwa Cho, Tae-Hoon Kim, Seung-Wan Hong, Eun-Hye Seo, Seong-Hyop Kim

**Affiliations:** 1 Department of Medicine, Konkuk University Graduate School, Seoul, Korea.; 2 Department of Orthopedic Surgery, Konkuk University Medical Center, Konkuk University School of Medicine, Seoul, Korea.; 3 Department of Anesthesiology and Pain medicine, Konkuk University Medical Center, Konkuk University School of Medicine, Seoul, Korea.; 4 Korea mRNA vaccine initiative, Gachon University, Songdo, Korea.; 5 Department of Medicine, Institute of Biomedical Science and Technology, Konkuk University School of Medicine, Seoul, Korea.

**Keywords:** frailty, postoperative cognitive dysfunction, glutathione peroxidase 4, threonine.

## Abstract

**Background:**

Based on previous studies showing that frailty is associated with postoperative adverse events in older patients undergoing various surgical procedures, older patients with frailty may be more vulnerable to POCD. This study was designed to evaluate the effect of frailty on postoperative cognitive dysfunction (POCD) in aged rats undergoing general anaesthesia.

**Methods:**

Male Sprague-Dawley rats aged 18 months were allocated to Control and Frailty groups according to hind limb immobilisation using aluminium splints. Before and after general anaesthesia, Y-maze test for cognitive function and frailty status were evaluated. Frailty, including muscle atrophy, assessed by quadriceps weight, was further evaluated by measuring glutathione peroxidase 4 (GPX4) and threonine.

**Results:**

Twenty rats were enrolled and evenly allocated between the two groups, with no dropouts. There was no significant difference in the alternation ratio in the Y maze test between the groups before general anaesthesia; however, a significant difference was observed two days after general anaesthesia (65% ± 37% in the Control group vs 29% ± 28% in the Frailty group, *p* = 0.01). Body weight was similar between the groups throughout the study. By contrast, quadriceps weight was significantly lower in the Frailty group [2.4 (2.3-2.6) g vs 2.0 (1.7-2.3) g, *p* = 0.0001]. GPX4 and threonine levels in the quadriceps were also significantly lower in the Frailty group.

**Conclusion:**

Frailty was significantly associated with POCD. Prevention of frailty may be important for reducing the risk of POCD.

## Introduction

The World Health Organization has emphasised that all countries are facing major challenges due to demographic shifts and should prepare for their impact on social systems, including the health system. This demographic shift is characterised by a 'much faster growing elderly population' 1. The increasing older adult population has also led to more frequent exposure to general anaesthesia for medical needs. One of the adverse events associated with general anaesthesia in older patients is postoperative cognitive dysfunction (POCD) 2. The mechanisms underlying POCD have recently been linked to neuroinflammation 3, 4, although they have not been fully elucidated 5.

Frailty is defined as an ageing-related syndrome of physiological decline, characterised by marked vulnerability to adverse health outcomes 6, 7. It is clinically common in the older population 8-10. Based on previous studies showing that frailty is associated with postoperative adverse events in older patients undergoing various surgical procedures 11, older patients with frailty may be more vulnerable to POCD.

The authors hypothesised that the occurrence of POCD may differ according to the presence of frailty in older individuals. This study was designed to evaluate the effect of frailty on POCD in aged rats undergoing general anaesthesia.

## Materials and Methods

All experiments were performed in accordance with the National Institutes of Health Guidelines for the Care and Use of Laboratory Animals. After obtaining approval from the Institutional Animal Care and Use Committee (IACUC) of Konkuk University (approval number: KU25108), all experiments were conducted at the Konkuk University Laboratory Animal Research Center in accordance with IACUC guidelines.

The study design is summarised in Figure 1.

### Experimental preparation

Male Sprague-Dawley rats aged 18 months were purchased from Koatech (Pyeongtaek, Korea). The rats were housed in cages with free access to water and food. The room was maintained on a standard 12-hour light/dark cycle, with lights on at 7:00 and off at 19:00, at a temperature of 25°C. The rats were acclimated to the experimental conditions for 7 days before the study and were fed a standard diet with free access to water.

### Body weight measurement

Body weight was measured before group allocation and on the two days following general anaesthesia.

### Cognitive function testing using Y-maze test 4, 12

After acclimation, the Y-maze test was performed to evaluate cognitive function immediately before group allocation and on the day before and two days after general anaesthesia. The Y-maze consisted of three arms (A, B, and C). Each arm had a length of 50 cm, height of 25 cm, and width of 10 cm and was connected to the other arms at an angle of 120°. Before the start of the test, all arms were wiped with 70% alcohol, and a board was placed at the centre of the Y-maze to close access to arm C. The rats were released from the cage at the end of arm A and allowed to move freely between arms A and B for 15 minutes to adapt to the device. After adaptation, the rats were returned to the cage and allowed to rest for 1 hour. Next, all arms were wiped again with 70% alcohol. The board blocking arm C was removed to allow free access to all arms, and the rats were released from the cage at the end of arm A. Movement was recorded using a video camera for 5 minutes. The total number of entries into each arm and the number of spontaneous alternations were recorded. The alternation ratio was calculated using the formula [(number of spontaneous alternations) / (total arm entries - 2)] × 100%. If a rat had an alternation ratio of < 40% on the day before group allocation, it was considered to have a pre-existing cognitive disorder and was excluded from the study.

### Frailty evaluation using wire hanging test

The wire hanging test was performed to evaluate frailty. This test is based on the instinct of rats to avoid falling. A transparent, internally visible hexahedron (length 54 cm, width 54 cm, height 80 cm) was prepared. The lid was replaced with a wire grid, and sufficient bedding was placed at the bottom to reduce shock when the rat fell. The test started when the rat was placed at the centre of the grid. After placement, the grid was turned upside down, and the time from when the rat began hanging on the grid to when its hind legs detached was measured. If a rat had a wire hanging time of < 1 second on the day before group allocation, it was considered to have a pre-existing neuromuscular disorder and was excluded from the study. The test was performed at the same time points as the Y-maze test and followed the Y-maze test. Rats in the Frailty group were tested without aluminium splints for the establishment of frailty.

### Experimental model with frailty

Frailty was induced by immobilisation of the hind legs using aluminium splints immediately after the first wire hanging test. The rats were randomly allocated to the Control group or the Frailty group according to immobilisation. After group allocation, anaesthesia was administered to both groups using oxygen (0.3 L/min) and nitrous oxide (0.7 L/min) in an induction chamber for 5 minutes. In the Frailty group, aluminium splints were applied to the hind legs and secured with an adhesive bandage to restrict hind limb movement during anaesthesia; this was not performed in the Control group. The splints were maintained for 7 days before general anaesthesia. During anaesthesia, blood samples were obtained from the right carotid artery in both groups. The samples were collected in ethylenediaminetetraacetic acid (EDTA) tubes (BD Vacutainer®, USA). The EDTA tubes were centrifuged at 1,500 × *g* at 4°C for 20 minutes to separate the plasma. The plasma was then transferred to 5-mL conical tubes and stored at -20°C. The rats were fed a standard diet with free access to water.

### General anaesthesia 4, 12-14

Anaesthesia was induced by intraperitoneal administration of ketamine (100 mg/kg; Yuhan, Korea) and xylazine (10 mg/kg; Sigma-Aldrich, USA). A heating pad was placed on the surgical platform to maintain the body temperature at approximately 37°C during anaesthesia. The rats were secured in the supine position and fastened to the surgical platform for endotracheal intubation using a 16 G catheter (length 45 mm; Dukwoo Medical, Korea). Correct placement of the catheter was confirmed by symmetrical chest expansion. The catheter was then connected to a ventilator (Harvard Apparatus, USA). The ventilator settings during anaesthesia were as follows: fraction of inspired oxygen, 0.5; inspired flow rate, 150 mL/min; tidal volume, 6 mL/kg; respiratory rate, 50 breaths/min; inspiration-to-expiration ratio, 1:1; and positive end-expiratory pressure, 5 cmH_2_O. After induction of anaesthesia, a blood sample was obtained from the right carotid artery and collected using the same procedure as described for anaesthesia after group allocation. Anaesthesia was maintained for 2 hours using isoflurane (1.5 vol%), after which the vaporiser was switched off. Mechanical ventilation was maintained until full recovery of spontaneous respiration was confirmed. The catheter was then removed, and the rats were returned to their cages.

### Brain and skeletal muscle preparation

After the Y-maze test on the second day after general anaesthesia, the rats were anaesthetised using isoflurane (5 vol%) with oxygen (0.3 L/min) and nitrous oxide (0.7 L/min) in an induction chamber. After confirmation of adequate anaesthesia, the rats were sacrificed. The abdomen was dissected to expose the abdominal aorta. A blood sample was obtained from the abdominal aorta and collected using the same procedure as that described for anaesthesia after group allocation. Following blood collection, 1× phosphate-buffered saline (PBS) was administered via the abdominal aorta until complete exsanguination was achieved and the liver colour changed from red to pale. Using scissors and forceps, the skull was opened and the brain was removed. The brain was divided into two hemispheres. The left hemisphere was prepared to assess neuronal damage and stored in a 50-mL conical tube containing 4% paraformaldehyde (PFA) (BIOSESANG, Korea). The right hemisphere was prepared to assess the inflammatory status, transferred into a 2-mL Eppendorf tube® (Eppendorf, Germany), and stored at -20°C. The quadriceps muscles were then obtained using scissors and forceps, and their weights were measured. The left quadriceps was prepared to assess muscle atrophy using haematoxylin and eosin staining and was stored in a 15-mL conical tube containing 4% PFA. The right quadriceps was prepared to assess frailty, transferred into a 5-mL Eppendorf tube®, and stored at -20°C. Neuronal damage in the brain was evaluated using immunofluorescence staining for caspase-1 as an indicator of apoptosis. Inflammatory status in the brain was assessed using enzyme-linked immunosorbent assay (ELISA) for cytokine detection. Frailty was evaluated using ELISA to measure glutathione peroxidase 4 (GPX4) and threonine levels in the quadriceps. Frailty was also assessed in blood samples using the same ELISA procedures.

### Neuronal damage in the brain

The stored left-brain hemisphere in the conical tube was cut into three pieces and transferred to paraffin cassettes. The cassettes containing the tissue were washed with water for 1 hour to remove 4% PFA. The washed cassettes were then transferred to a tissue processor (Leica Biosystems, Germany) for paraffin processing. The processing programme was as follows: formalin 1 for 1 hour, formalin 2 for 1 hour, 70% ethanol for 1 hour, 80% ethanol for 1 hour, 90% ethanol for 1 hour, 100% ethanol (twice) for 1 hour each, xylene (twice) for 1 hour each, and paraffin (twice) for 2 hours each.

After processing, the cassettes were transferred to a tissue embedding centre (Leica Biosystems) for paraffin embedding, then placed in a freezer for 30 minutes to solidify the blocks. Tissue sections were cut using a microtome (Leica Biosystems) and floated in a warm water bath at 37°C. The sections were mounted onto microscope slides and dried on a hotplate at 40°C for 1 hour, followed by 56°C for 30 minutes. The mounted sections were immersed in xylene (two changes; 15 and 10 minutes) to remove paraffin, and then rehydrated through graded ethanol (100% twice, 90%, 80%, and 70%; 3 minutes each). Antigen retrieval was performed in 1× citrate buffer, with heating in a microwave for 5 minutes, repeated three times. The sections were cooled at room temperature for 20 minutes and then blocked with 5% goat serum.

After blocking, the sections were washed with 1× PBS for 5 minutes (three times). A caspase-1 antibody (Invitrogen, USA) was used as the primary antibody for neuronal damage, diluted 1:200 in 5% goat serum. The sections were incubated with the primary antibody at room temperature for 1 hour, followed by washing with 1× PBS (5 minutes, three times). A 488 anti-rabbit antibody (Invitrogen) was used as the secondary antibody, diluted 1:1,000 in 5% goat serum. The sections were incubated with the secondary antibody in the dark at room temperature for 1 hour, then washed with 1× PBS (5 minutes, three times).

For nuclear staining, 4′,6-diamidino-2-phenylindole (DAPI) (Invitrogen) was used, diluted 1:2,000 in distilled water. The sections were incubated with DAPI in the dark at room temperature for 5 minutes, followed by washing with 1× PBS (5 minutes, three times).

The sections were then dehydrated through graded ethanol (70%, 80%, 90%, and 100% twice; 3 minutes each), followed by xylene (twice; 3 minutes each). After staining, the sections were mounted with a coverslip using anti-fade mounting medium (Vector, USA). The coverslips were sealed with nail polish. The stained sections were observed using a fluorescence microscope (Olympus, Japan).

### Inflammatory status in the brain 4, 12

Pro-inflammatory cytokines, including tumour necrosis factor-α (TNF-α) (Abcam, USA) and interleukin (IL)-1β (Abcam), as well as anti-inflammatory cytokines (IL-4 and IL-10), were measured to assess the inflammatory status in the brain. The stored right hemisphere was homogenised and centrifuged at 12,000 × *g* for 15 minutes at 4°C. The supernatant was collected and used for cytokine detection using ELISA kits. Cytokine levels were measured using a microplate reader.

### Evaluation of muscle atrophy in the quadriceps using haematoxylin and eosin staining

The quadriceps muscle was cut perpendicularly at the centre, and the cross-sectional area was measured 15.

### Frailty assessment using ELISA for GPX4 and threonine in the quadriceps

GPX4 and threonine levels in the quadriceps were measured to assess frailty. The stored quadriceps tissue was homogenised and centrifuged at 12,000 × *g* for 15 minutes at 4°C. The supernatant was used to detect GPX4 and threonine using ELISA kits. Threonine was measured together with serine because no ELISA kit specific for threonine in rats was available. The levels were read using a microplate reader.

### Frailty assessment using ELISA for GPX4 and threonine in the blood

The same procedure was used for blood samples as that described for the quadriceps. Threonine was measured together with serine for the same reason.

### Statistical analysis

The primary outcome was the alternation ratio in the Y-maze test on day 2 after general anaesthesia, compared between the two groups. A pilot study was performed using three rats in the Frailty group and three in the Control group. The pilot study showed alternation ratios of 31% ± 27% in the Frailty group and 74% ± 27% in the Control group. Based on these results, a sample size of 10 rats per group was calculated to achieve a power of 0.9 with an α value of 0.05.

Intra-group and inter-group differences were analysed with the unpaired t-test, Mann-Whitney U test, and two-way repeated-measures analysis of variance, using GraphPad Prism software (version 5.01; GraphPad Software, La Jolla, CA, USA). A p-value of < 0.05 was considered statistically significant. Data are presented as mean ± standard deviation or median (interquartile range).

## Results

Twenty rats were enrolled in the study and evenly allocated into the two groups, with no dropouts.

The alternation ratios of the two groups, assessed using the Y-maze test, were similar before general anaesthesia (immediately before group allocation: 69% ± 14% in the Control group vs 77% ± 17% in the Frailty group, *p* = 0.74; on the day before general anaesthesia: 68% ± 22% vs 73% ± 29%, *p* = 1.00). However, a significant difference was observed two days after general anaesthesia (65% ± 37% in the Control group vs 29% ± 28% in the Frailty group, *p* = 0.01). The Frailty group showed a significant decline in the alternation ratio after general anaesthesia (Figure 2).

Wire hanging test performance was similar between the groups immediately before group allocation (3.7 ± 1.3 seconds in the Control group vs 3.9 ± 1.2 seconds in the Frailty group, *p* = 0.59). The Frailty group showed a significant decline in performance after immobilisation of the hind legs via aluminium splints (the day before general anaesthesia: 4.4 ± 2.2 seconds in the Control group vs 1.9 ± 1.1 seconds in the Frailty group, *p* = 0.01; two days after general anaesthesia: 4.4 ± 1.1 seconds vs 1.3 ± 1.1 seconds, *p* = 0.02). However, there was no significant difference between the day before and two days after general anaesthesia within the Frailty group (*p* = 0.57) (Figure 3).

Neuronal damage, assessed by caspase-1 expression, was significantly higher in the Frailty group. The expression level of caspase-1 was increased in this group (2.84% ± 0.68% in the Control group vs 10.28% ± 2.72% in the Frailty group, *p* < 0.0001) (Figure 4A). The number of caspase-1-positive cells was also significantly higher in the Frailty group (47.70% ± 16.06% vs 227.00% ± 50.12%, *p* < 0.001) (Figure 4B).

The pro-inflammatory cytokines TNF-α and IL-1β were significantly higher in the Frailty group than in the Control group, whereas the anti-inflammatory cytokines IL-10 and IL-4 were significantly lower in the Frailty group (Table 1).

Body weight was similar between the two groups throughout the study. However, quadriceps weight was significantly lower in the Frailty group than in the Control group (2.6 ± 0.4 g vs 2.1 ± 0.4 g, *p* = 0.003) (Table 2). The cross-sectional area of the quadriceps was also significantly lower in the Frailty group (14.8 ± 2.8 cm^2^ in the Control group vs 7.00 ± 0.8 cm^2^ in the Frailty group, *p* = 0.0001) (Table 2).

The GPX4 and threonine levels in the quadriceps were significantly lower in the Frailty group than in the Control group (Table 3).

Blood GPX4 levels were similar between the two groups immediately before group allocation (203.80 ± 50.28 pg/mL in the Control group vs 249.20 ± 13.44 pg/mL in the Frailty group, *p* = 0.73). The Frailty group showed a significant decline in GPX4 levels after immobilisation (the day before general anaesthesia: 209.00 ± 36.56 pg/mL in the Control group vs 43.50 ± 46.67 pg/mL in the Frailty group, *p* = 0.003; two days after general anaesthesia: 221.7 ± 25.44 pg/mL vs 46.97 ± 35.76 pg/mL, *p* < 0.0001). However, there was no significant difference between the day before and two days after general anaesthesia within the Frailty group (*p* = 0.99) (Figure 5A).

Changes in blood threonine levels showed a similar pattern to that of GPX4 in the two groups. However, the Control group showed a significant decline after general anaesthesia, although threonine levels remained significantly higher than in the Frailty group (Figure 5B).

## Discussion

The present study showed that the Frailty group, characterised by a significant decline in performance in the wire hanging test and significantly lower levels of frailty-related biomarkers (GPX4 and threonine) in the quadriceps and blood, was more vulnerable to POCD than the Control group.

Although the definition and mechanisms of frailty have not been fully established, oxidative stress and inflammation have been closely associated with frailty 6, 16. Accordingly, related biomarkers have been used to evaluate frailty. In the present study, GPX4 and threonine were used for this purpose. The basic mechanism of the ageing process has traditionally been described by the 'free radical theory' 17, which proposes that ageing results from the accumulation of oxidative damage caused by reactive oxygen species (ROS) within cells 16, 18. Since 2000, this concept has evolved, and ageing is now also considered a consequence of asymptomatic, chronic, low-grade inflammation 19, 20. More recently, imbalance in iron metabolism has been recognised as a key contributor to the ageing process 21, 22. GPX4 regulates ROS-induced cell death and plays a central role in ferroptosis, an iron-dependent, non-apoptotic form of cell death 23-25. By inhibiting ferroptosis, GPX4 contributes to cell survival 26. Threonine also plays a critical role in cell maintenance and renewal 27. As an essential amino acid, it is required for protein biosynthesis, and increased threonine levels have been associated with reduced ROS production and inhibition of ferroptosis 28. Increased ROS and inflammation contribute to the ageing process, ultimately leading to the production of pro-inflammatory cytokines 29. Consistent with these mechanisms, the Frailty group in the present study showed significantly higher levels of pro-inflammatory cytokines and lower levels of anti-inflammatory cytokines than the Control group. Therefore, GPX4 and threonine were evaluated as biomarkers of frailty in this study.

The findings for GPX4 in the blood, unlike those for threonine, were particularly notable in the present study. After the establishment of frailty, the levels of both GPX4 and threonine in the blood were significantly decreased, suggesting that these markers are suitable for evaluating frailty. However, the level of GPX4 in the blood did not change before and after general anaesthesia, whereas threonine levels decreased significantly after general anaesthesia. This suggests that the effect of general anaesthesia may be limited for GPX4, but not for threonine. Therefore, GPX4 in the blood may serve as a biomarker for frailty independently of general anaesthesia, although further studies are needed to confirm this.

In the present study, frailty was established by immobilisation of the hind legs. Animal models of frailty can be generated using genetic or non-genetic approaches 30, 31. To induce physical decline, restriction of movement is a recognised method for modelling frailty 32. Several studies have shown that voluntary exercise delays the ageing process, including frailty, whereas reduced activity accelerates it 32-34. Based on this, frailty was induced by hind limb immobilisation in the present study. However, this approach has some limitations. First, there is a possibility that forelimb muscle strength increased as a compensatory mechanism in the Frailty group. The similar body weight between groups, despite a significant difference in quadriceps weight, may reflect such compensation, although forelimb muscle mass was not measured. Nevertheless, the clear difference in wire hanging test performance between the groups suggests that any compensatory effect of the forelimbs was limited. Second, the procedure used to establish frailty was performed under anaesthesia, and the effects of anaesthesia itself may have influenced the results. To minimise this confounding factor, anaesthesia was administered to both the Frailty and Control groups, even though it was not required for the Control group.

In the present study, the wire hanging test was used to evaluate frailty. This behavioural test assesses neuromuscular function and strength, and a decline in hanging performance indicates neuromuscular impairment. It therefore reflects physical activity and was used as a tool for evaluating frailty.

In the present study, cognitive function was assessed using the Y-maze test alone. Although a single behavioral test may not fully capture the multidimensional aspects of cognitive function, there are currently no universally accepted diagnostic criteria or cutoff values for a specific cognitive test to define postoperative cognitive POCD, and substantial heterogeneity exists in the criteria used to define postoperative cognitive decline. Nevertheless, the Y-maze spontaneous alternation test is a widely used behavioral method for assessing spatial working memory and short-term cognitive function in rodents 35, 36. Moreover, previous studies investigating postoperative cognitive impairment in rodent models have also used the Y-maze test to evaluate postoperative cognitive function 4, 12. Therefore, the Y-maze test provides an appropriate and well-established behavioral measure for assessing cognitive changes in this experimental model, although the findings should be interpreted in consideration of the use of a single cognitive assessment. Similarly, there is no universally accepted absolute cutoff value for individual physical performance measures used to characterize frailty in rodents. Previous rodent studies have used cohort-specific criteria for physical performance measures, including the wire-hanging test 37, 38. Therefore, the criterion of a wire-hanging time of < 1 second used in the present study was based on previously reported experimental approaches and was applied to exclude rats with apparent pre-existing neuromuscular impairment, rather than to define frailty using a universal diagnostic threshold 39, 40.

In conclusion, frailty reduced physical activity and muscle weight, although body weight remained unchanged. Frailty was significantly associated with POCD. Prevention of frailty may therefore be important for reducing the risk of POCD.

## Figures and Tables

**Figure 1:**
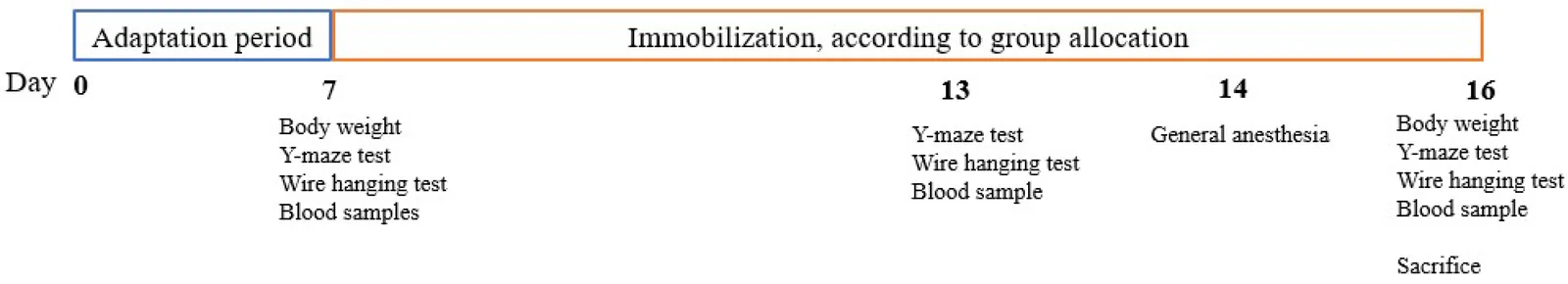
Summary of study design.

**Figure 2:**
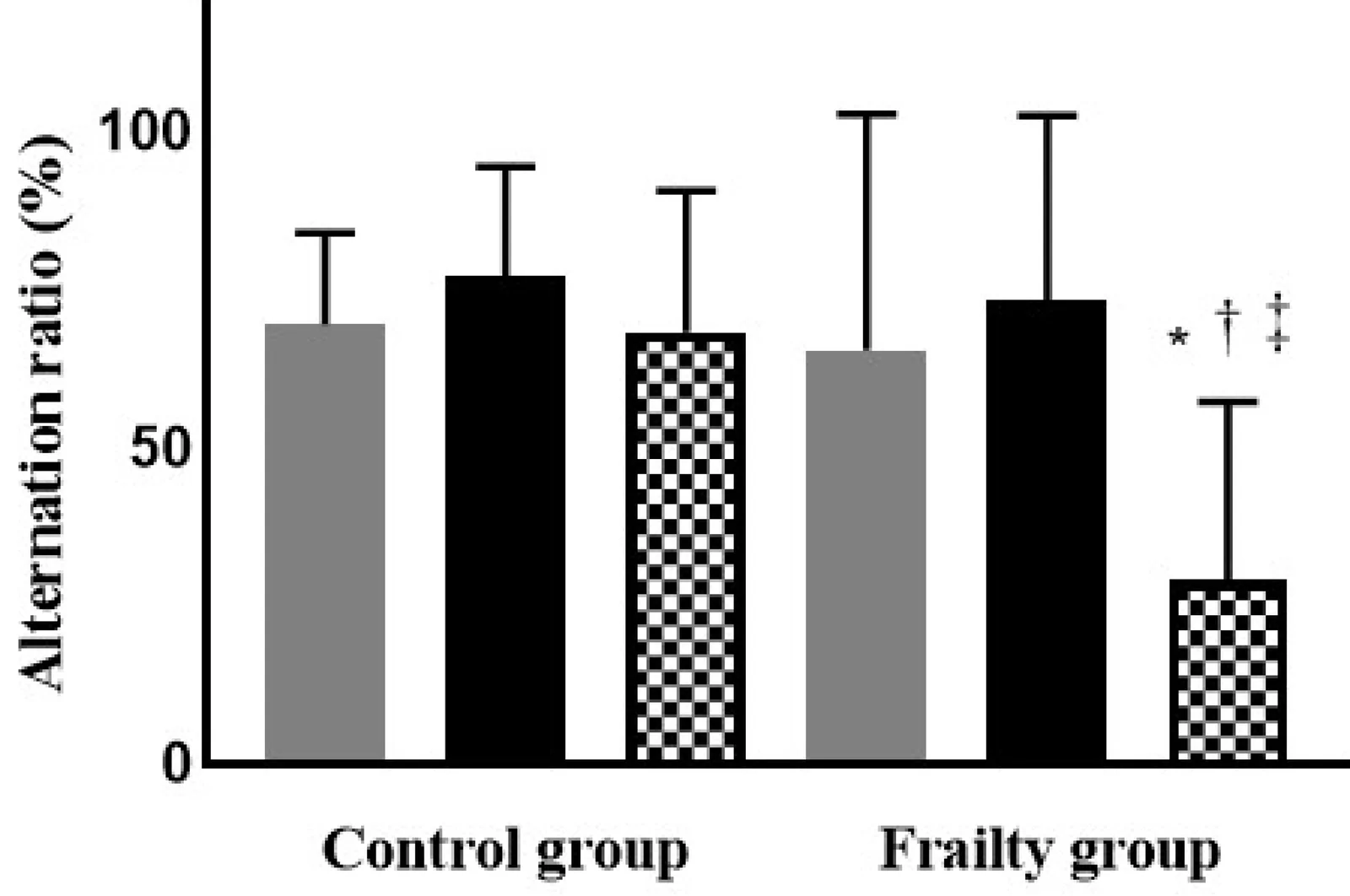
The change of cognitive function, using Y-maze test just before group allocation (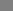), and on the day just before (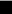) and two days after general anesthesia (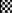). ^*^: *p* < 0.05, compared with Control group. ^†^:* p* < 0.05, compared with just before group allocation. ^‡^: *p* < 0.05, compared with the day just before general anesthesia.

**Figure 3:**
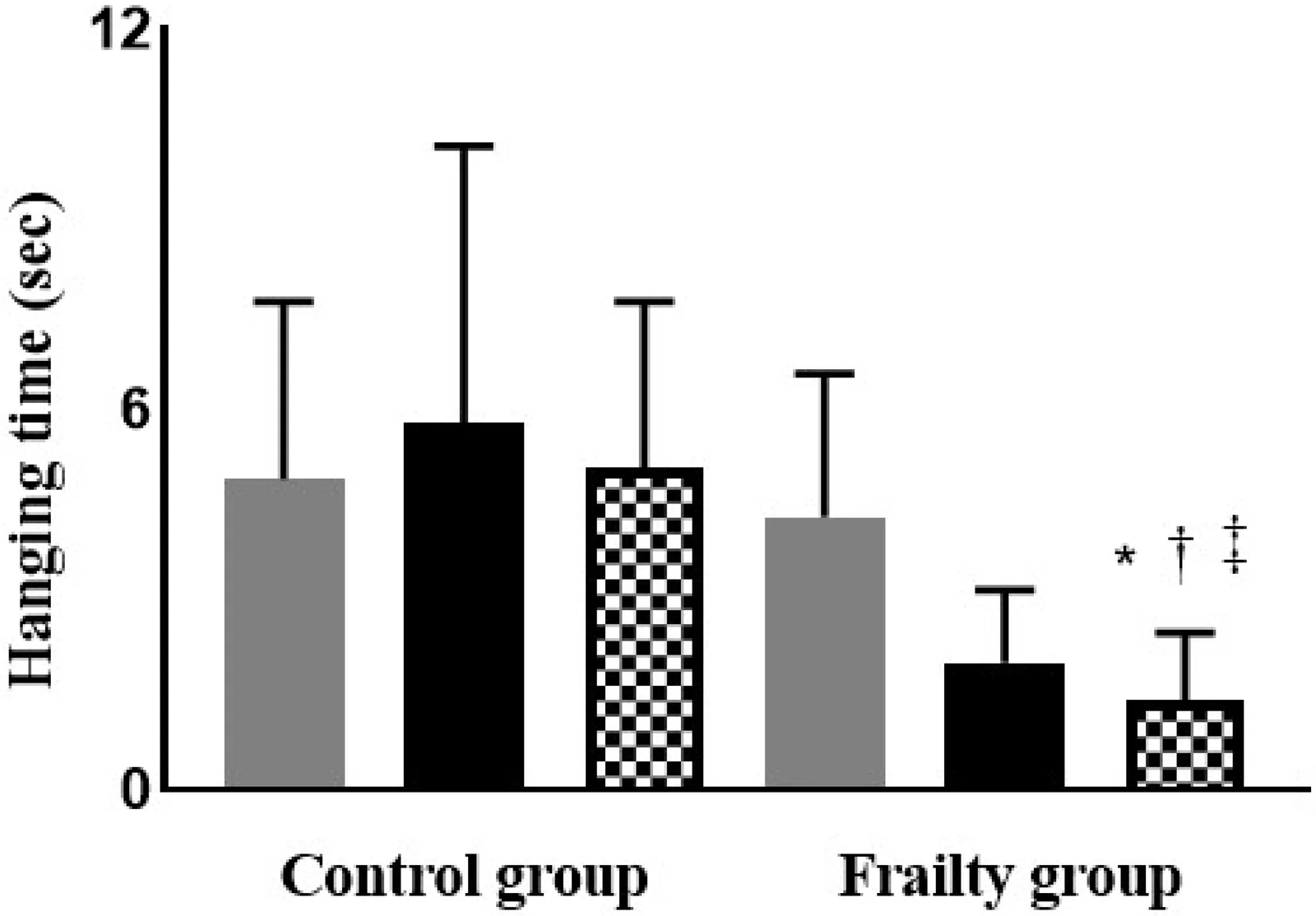
The change of frailty, using wire hanging test just before group allocation (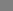), and on the day just before (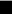) and two days after general anesthesia (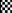). ^*^: *p* < 0.05, compared with Control group. ^†^:* p* < 0.05, compared with just before group allocation. ^‡^: *p* < 0.05, compared with the day just before general anesthesia.

**Figure 4:**
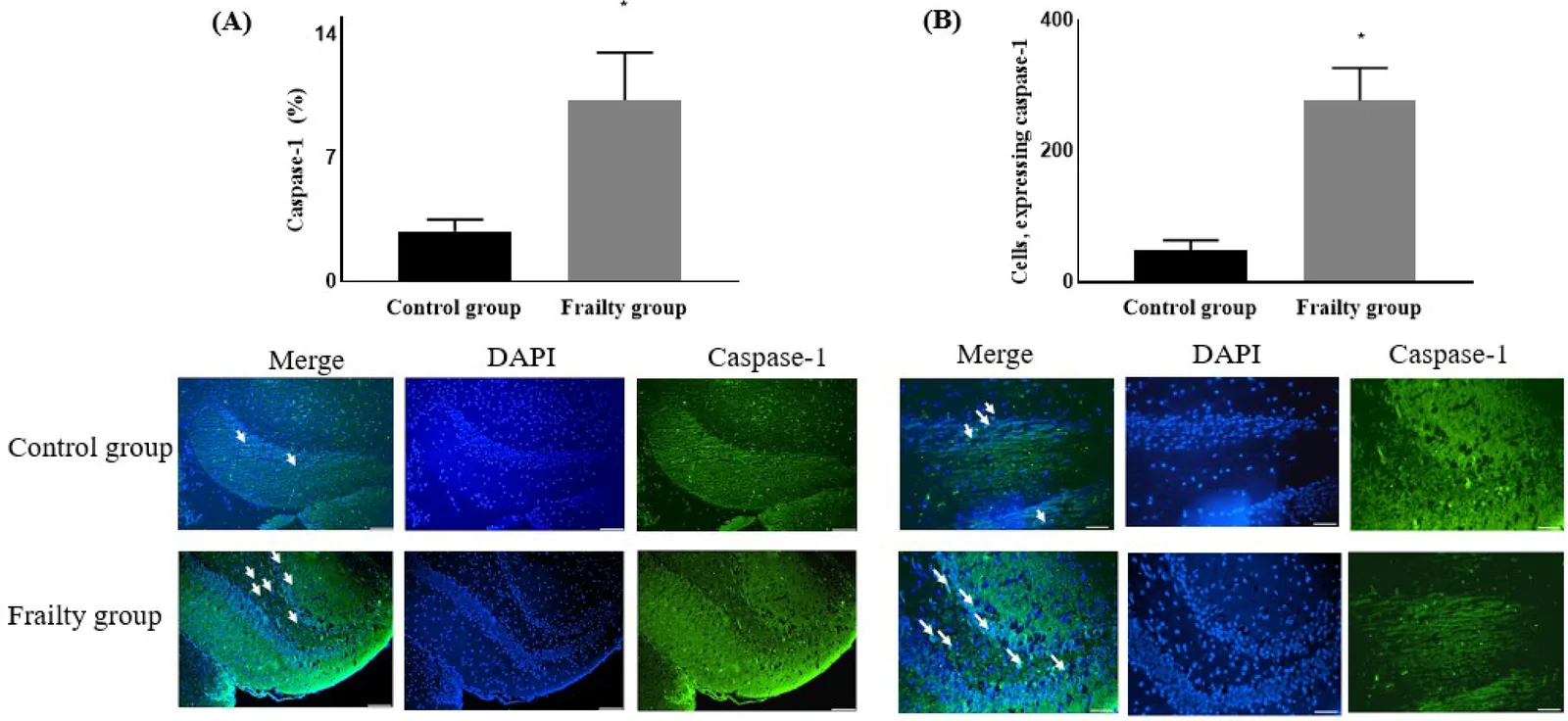
Neuronal damage, using the expression of caspase-1 (represented with white arrows) (A) and the number of cells, expressing caspase-1 (represented with white arrows) (B). ^*^: *p* = 0.0001, compared with Control group. **Abbreviations:** DAPI, 4′,6-diamidino-2-phenylindole.

**Figure 5:**
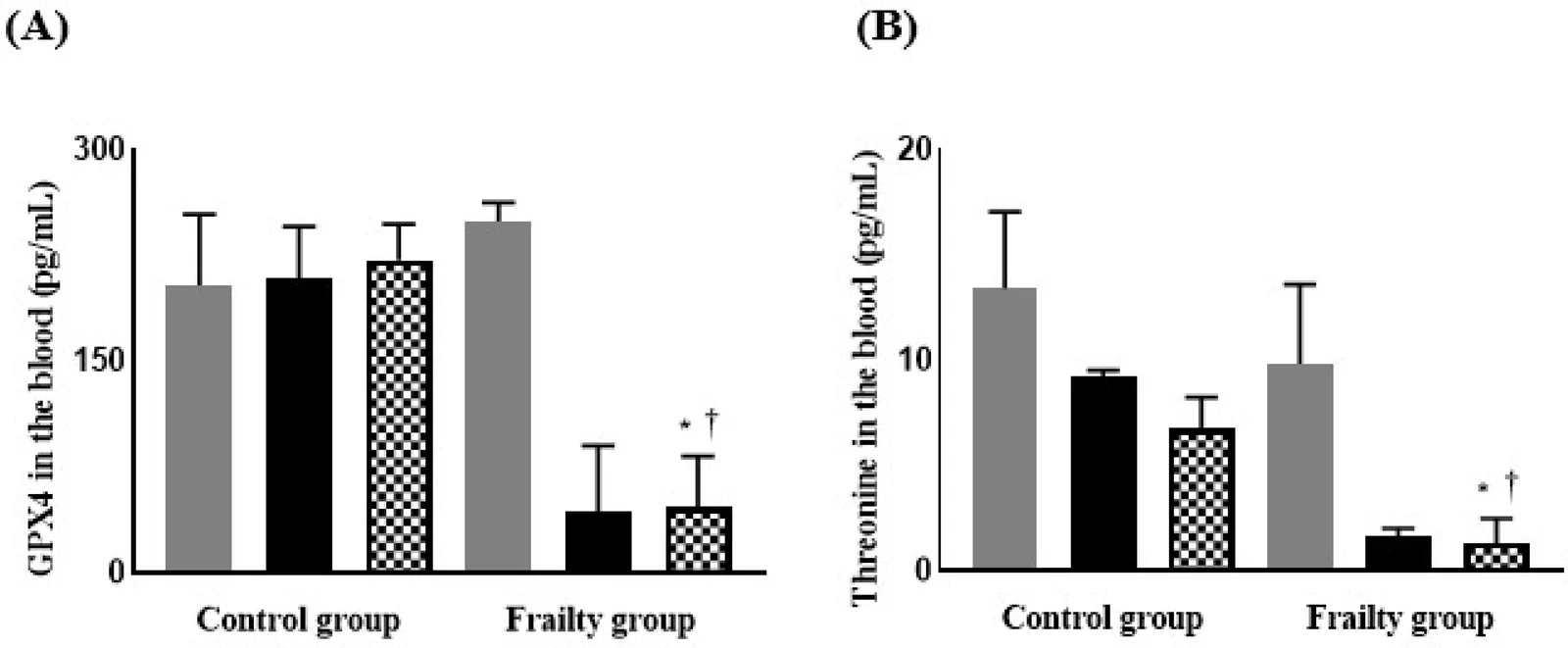
The change of glutathione peroxidase 4 (GPX4) (A) and threonine (B) in the blood. ^*^: *p* < 0.05, compared with Control group. ^†^:* p* < 0.05, compared with just before group allocation.

**Table 1:** Enzyme-linked immunosorbent assay (ELISA) for the detection of cytokines in the brain between Control group and Frailty group.

	Control group	Frailty group	*p* value
Pro-inflammatory cytokines			
TNF-α (pg/ml)	77.72 ± 9.34	102.40 ± 4.05	0.0001
IL-1β (pg/ml)	41.75 ± 15.02	143.10 ± 20.59	0.0001
Anti-inflammatory cytokines			
IL-4 (pg/ml)	198.30 (190.20-199.20)	185.00 (170.00-188.80)	0.0005
IL-10 (pg/ml)	82.59 ± 9.56	42.91 ± 8.25	0.0001

Data is expressed as mean ± standard deviation or median (interquartile range). **Abbreviations:** TNF, tumor necrosis factor; IL, interleukin.

**Table 2:** Body weight, both quadriceps weight and cross-sectional area (CSA) of the quadriceps between Control group and Frailty group.

	Control group	Frailty group	*p* value
Body weight (g)			
Just before group allocation	263 (233-254)	247 (228-243)	0.27
On the day just before general anesthesia	-	-	-
Two days after general anesthesia	325 (240-284)	313 (230-255)	0.28
Quadriceps weights (g)			
Both	2.4 (2.3-2.6)	1.95 (1.7-2.3)	0.0001
Right	2.3 ± 1.7	1.9 ± 0.4	0.003
Left	2.6 ± 0.4	2.1 ± 0.4	0.003
CSA of quadriceps (cm^2^)	14.8 ± 2.8	7.0 ± 0.8	0.0001

Data is expressed as median (interquartile range) or mean ± standard deviation.

**Table 3:** Enzyme-linked immunosorbent assay (ELISA) for the detection of glutathione peroxidase 4 (GPX4) and threonine in the quadriceps between Control group and Frailty group.

	Control group	Frailty group	*p* value
GPX4 (pg/mL)	304.7 (257.4-315.8)	114.1 (24.53-195.6)	0.0001
Threonine (pg/mL)	20.1 (12.1-21.9)	6.0 (4.1-6.5)	0.0001

Data is expressed as median (interquartile range) or mean ± standard deviation.

## Data Availability

The data that support the findings of this study are available from the corresponding author upon reasonable request.

## References

[1] Khan HT, Addo KM, Findlay H (2024). Public health challenges and responses to the growing ageing populations. Public Health Challenges.

[2] Amirfarzan H, Schumann R, Leissner KB (2025). Anesthesia and Sedation in Older Adults with Pre-existing Cognitive Impairment. Drugs & Aging.

[3] Peng W, Lu W, Jiang X, Xiong C, Chai H, Cai L (2023). Current progress on neuroinflammation-mediated postoperative cognitive dysfunction: an update. Current Molecular Medicine.

[4] Cho E-H, In C-B, Lee G-W, Hong S-W, Seo E-H, Lee WH (2024). The preventive effect of urinary trypsin inhibitor on postoperative cognitive dysfunction, on the aspect of behavior, evaluated by Y-maze test, via modulation of microglial activity. International Journal of Molecular Sciences.

[5] Li Z, Zhu Y, Kang Y, Qin S, Chai J (2022). Neuroinflammation as the underlying mechanism of postoperative cognitive dysfunction and therapeutic strategies. Frontiers in Cellular Neuroscience.

[6] Sato R, Vatic M, Peixoto da Fonseca GW, Anker SD, von Haehling S (2024). Biological basis and treatment of frailty and sarcopenia. Cardiovascular research.

[7] Perazza LR, Brown-Borg HM, Thompson LV (2022). Physiological systems in promoting frailty. Comprehensive Physiology.

[8] Chen X, Mao G, Leng SX (2014). Frailty syndrome: an overview. Clinical interventions in aging.

[9] Sieber CC (2017). Frailty-from concept to clinical practice. Experimental gerontology.

[10] Kim DH, Rockwood K (2024). Frailty in older adults. New England Journal of Medicine.

[11] Lin H-S, Watts J, Peel N, Hubbard R (2016). Frailty and post-operative outcomes in older surgical patients: a systematic review. BMC geriatrics.

[12] Cho E-H, Seo E-H, Hong S-W, Kim S-H (2024). The Preventive Effect of Ulinastatin on Blood-Brain Barrier Dysfunction in Rats with Postoperative Cognitive Dysfunction After General Anaesthesia with Isoflurane. International Journal of Molecular Sciences.

[13] Piao L, Na O-H, Seo E-H, Hong S-W, Sohn K-M, Kwon Y (2022). Effects of general anaesthesia with an inhalational anaesthetic agent on the expression of exosomes in rats. International Journal of Medical Sciences.

[14] Seo E-H, Piao L, Cho E-H, Hong S-W, Kim S-H (2023). The effect of ketamine on endoplasmic reticulum stress in rats with neuropathic pain. International Journal of Molecular Sciences.

[15] Yagi M, Taniguchi M, Tateuchi H, Hirono T, Fukumoto Y, Yamagata M (2022). Age-and sex-related differences of muscle cross-sectional area in iliocapsularis: a cross-sectional study. BMC geriatrics.

[16] El Assar M, Angulo J, Rodríguez-Mañas L (2020). Frailty as a phenotypic manifestation of underlying oxidative stress. Free Radical Biology and Medicine.

[17] Polidori MC, Mecocci P (2022). Modeling the dynamics of energy imbalance: The free radical theory of aging and frailty revisited. Free Radical Biology and Medicine.

[18] Jomova K, Raptova R, Alomar SY, Alwasel SH, Nepovimova E, Kuca K (2023). Reactive oxygen species, toxicity, oxidative stress, and antioxidants: chronic diseases and aging. Archives of toxicology.

[19] Bruunsgaard H (2006). The clinical impact of systemic low-level inflammation in elderly populations. Dan Med Bull.

[20] Hubbard RE, Woodhouse KW (2010). Frailty, inflammation and the elderly. Biogerontology.

[21] Alves FM, Ayton S, Bush AI, Lynch GS, Koopman R (2023). Age-related changes in skeletal muscle iron homeostasis. The Journals of Gerontology: Series A.

[22] Tian Y, Tian Y, Yuan Z, Zeng Y, Wang S, Fan X (2022). Iron metabolism in aging and age-related diseases. International journal of molecular sciences.

[23] Wang B, Wang Y, Zhang J, Hu C, Jiang J, Li Y (2023). ROS-induced lipid peroxidation modulates cell death outcome: mechanisms behind apoptosis, autophagy, and ferroptosis. Archives of toxicology.

[24] Li J, Cao F, Yin H-l, Huang Z-j, Lin Z-t, Mao N (2020). Ferroptosis: past, present and future. Cell death & disease.

[25] Endale HT, Tesfaye W, Mengstie TA (2023). ROS induced lipid peroxidation and their role in ferroptosis. Frontiers in cell and Developmental Biology.

[26] Xie Y, Kang R, Klionsky DJ, Tang D (2023). GPX4 in cell death, autophagy, and disease. Autophagy.

[27] Tang Q, Tan P, Ma N, Ma X (2021). Physiological functions of threonine in animals: beyond nutrition metabolism. Nutrients.

[28] Kim J, Jo Y, Cho D, Ryu D (2022). L-threonine promotes healthspan by expediting ferritin-dependent ferroptosis inhibition in C. elegans. Nature Communications.

[29] Nafe R, Hattingen E (2024). Forms of non-apoptotic cell death and their role in gliomas-presentation of the current state of knowledge. Biomedicines.

[30] Liu P, Li Y, Ma L (2022). Frailty in rodents: Models, underlying mechanisms, and management. Ageing Research Reviews.

[31] Pignolo RJ, Chandra A (2025). Insights into age-related osteoporosis from senescence-based preclinical models and human accelerated aging paradigms. Mechanisms of Ageing and Development.

[32] Merchant R, Morley J, Izquierdo M (2021). Exercise, aging and frailty: guidelines for increasing function. The journal of nutrition, health & aging.

[33] Vina J, Rodriguez-Mañas L, Salvador-Pascual A, Tarazona-Santabalbina FJ, Gomez-Cabrera MC (2016). Exercise: the lifelong supplement for healthy ageing and slowing down the onset of frailty. The Journal of physiology.

[34] Caruso MG, Nicolas S, Lucassen PJ, Mul JD, O'Leary OF, Nolan YM (2024). Ageing, cognitive decline, and effects of physical exercise: complexities, and considerations from animal models. Brain Plasticity.

[35] Kraeuter A-K, Guest PC, Sarnyai Z (2018). The Y-maze for assessment of spatial working and reference memory in mice. Pre-clinical models: Techniques and protocols: Springer.

[36] KSV ABG, Dissanayake D, Gunatilake M, Velu VK, Paranthaman M (2023). A short review on behavioural assessment methods in rodents. Bioinformation.

[37] Baumann CW, Kwak D, Thompson LV (2020). Phenotypic frailty assessment in mice: development, discoveries, and experimental considerations. Physiology.

[38] Hoffman E, Winder SJ (2016). A modified wire hanging apparatus for small animal muscle function testing. PLoS currents.

[39] Lv S, Zhang X, Feng Y, Zhou Y, Cui B, Yang Y (2020). Intravenous administration of pyroglutamyl apelin-13 alleviates murine inflammatory pain via the kappa opioid receptor. Frontiers in Neuroscience.

[40] Klein SM, Vykoukal J, Lechler P, Zeitler K, Gehmert S, Schreml S (2012). Noninvasive in vivo assessment of muscle impairment in the mdx mouse model-a comparison of two common wire hanging methods with two different results. Journal of neuroscience methods.

